# Routine-Deviation Detection in Smart-Home Sensor Networks Using GRU Prediction

**DOI:** 10.3390/s26144463

**Published:** 2026-07-14

**Authors:** Abeer Aman, Rashmi Kumari, Raja Omman Zafar, Yves Rybarczyk

**Affiliations:** School of Information and Engineering, Dalarna University, 791 88 Falun, Sweden; v25abeam@du.se (A.A.); v25rakum@du.se (R.K.); roz@du.se (R.O.Z.)

**Keywords:** sensor networks, routine deviation, anomaly detection, behavioral monitoring, Gated Recurrent Unit, routine prediction, decision support

## Abstract

**Highlights:**

**What are the main findings?**

- Individualization of Behavioral Routines: Optimal behavioral baselines differ significantly among individuals, with single-zone sensor configurations consistently outperforming multi-zone combinations by eliminating cumulative behavioral noise.
- Temporal Patterns of Candidate Routine Deviations: Behavioral deviations in smart homes are primarily characterized by precise hourly temporal shifts and localized activity spikes throughout the day, rather than simple uniform changes in total daily activity volume.

**What are the implications of the main findings?**

- Shift to Personalized Ambient Care: Anomaly detection systems must prioritize personalized, resident-specific baselines over globally applied population thresholds to minimize false alarms and adapt to individual lifestyle heterogeneity.
- Human-in-the-Loop Support for Interpreting Candidate Routine Deviations: Learning normal routines directly from unlabeled, non-intrusive sensor data allows for automated screening that seamlessly integrates with visual analytics, offering a proof-of-concept explainable pipeline for future home-care digital twins.

**Abstract:**

Smart-home sensor networks enable unobtrusive monitoring of daily activity and are increasingly used to support independent living among older adults. However, many anomaly detection methods produce scalar anomaly scores or binary alerts without explaining how the detected behavior differs from a resident’s normal routine. This paper proposes a two-stage framework for interpretable routine-deviation assessment using smart-home motion and door-contact sensors. In Stage 1, raw sensor streams are aligned on a two-second master calendar, aggregated into hourly event counts, mapped into functional household activity zones, and converted into daily routine profiles. A Gated Recurrent Unit (GRU) routine prediction model is trained using a three-day lookback window to predict expected daily zone-level activity. Candidate routine-deviation days are automatically identified from daily prediction errors. In Stage 2, the recent monitoring period is plotted as 24-h radar profiles against the learned routine model, allowing a human expert to visually assess deviations in timing, location, and severity. The workflow was evaluated using 28 days of smart-home data collected from multiple independent residents. The proposed GRU framework achieved RMSE values ranging from 0.136 to 0.180 and MAE values ranging from 0.126 to 0.138 across the four participants, consistently outperforming the Previous-Day Baseline and generally providing lower prediction errors than the Seasonal Naïve Baseline. These findings demonstrate the effectiveness of participant-specific routine modeling for personalized routine-deviation detection in smart-home environments. The results indicate that deviation-sensitive target zones differed across the four participants, suggesting the importance of participant-specific routine modeling. The proposed approach successfully links automated candidate routine-deviation identification with radar-based visual analytics, providing a proof-of-concept, personalized, and interpretable decision support workflow for ambient assisted living research.

## 1. Introduction

Population aging is one of the most significant demographic changes in the twenty-first century. The United Nations World Population Prospects 2024 report estimates that the proportion of people aged 65 years and above will increase from 10.3 per cent of the global population in 2024 to 20.7 per cent by 2080, eventually overtaking the number of children under 18 years of age; the World Health Organization similarly estimates that the global population aged 60 years and older will grow from approximately one billion in 2020 to 1.4 billion by 2030, with a projected doubling by 2050. These demographic changes are increasing demand for scalable and sustainable healthcare technologies that can support independent living, early risk detection and remote health monitoring for older adults [1,2]. In this context, smart-home sensor networks have been widely recognized as promising tools for aging in place and ambient assisted living because they enable continuous, unobtrusive monitoring of everyday behavior in residential environments [3,4,5]. Passive motion sensors, contact sensors, and environmental sensing devices are particularly suitable for this purpose because they can capture movement, room occupancy, object interaction, entrance door use, and daily household routines without requiring continuous user interaction or camera-based observation [6,7,8]. Compared with video-based monitoring, ambient sensing offers a more privacy-preserving approach while still providing meaningful behavioral indicators that can support routine modeling and anomaly detection in smart-home healthcare applications [9,10].

Behavioral changes inside the home may reflect emerging health concerns, functional decline, altered sleep patterns, reduced mobility, changes in activities of daily living, or deviations from established household routines. Previous smart-home studies have shown that sensor-derived behavior can support activity-quality assessment, cognitive and physical health monitoring, health-event detection, and routine discovery. Ref. [11] demonstrated that smart-home systems can assess the quality of activities performed in residential environments, while Ref. [12] showed that smart-home behavior data can support automated clinical assessment. Ref. [13] further demonstrated that changes in smart-home sensor patterns can support the detection and analysis of health-related events. However, translating raw sensor activations into clinically meaningful anomaly information remains challenging. Sensor events are sparse, indirect, user-specific, and highly dependent on contextual factors. A fixed rule that works for one resident may generate false alerts for another because normal routines differ substantially across individuals [9].

Recent anomaly detection research has increasingly moved toward deep learning, edge intelligence, non-wearable sensing, synthetic data generation, and personalized modeling. Ref. [14] addressed the shortage of real anomaly data by proposing sensor data simulation for anomaly detection among older adults living alone. Ref. [15] introduced a graph neural network approach for adverse health detection and personalized decision-making in sensor-based remote monitoring for dementia care. Ref. [7] proposed an IoT and edge-intelligence framework for monitoring older adults using anomaly detection on data from non-wearable sensors. Ref. [8] investigated non-intrusive binary sensors for monitoring and detecting mobility loss in older adults. Ref. [10] developed a smart-home-assisted anomaly detection system for older adults using deep learning and a comprehensive set of daily activities. These recent studies demonstrate strong progress in smart-home anomaly detection, but they also highlight persistent challenges related to limited anomaly labels, personalization, interpretability, and practical decision support.

Although notable progress has been made, important gaps in the existing research still persist. First, smart-home datasets collected from older adults are frequently unlabeled, making fully supervised anomaly detection difficult to apply in real-world residential monitoring. Second, many existing approaches prioritize automatic detection performance while providing limited explanation of why an anomaly was detected, which part of the home contributed to the alert, or when the deviation occurred during the day. Third, relatively few studies integrate personalized routine modeling, automatic candidate anomaly detection, and interpretable visualization within a single framework designed to support human decision-making. Addressing these gaps is essential for the practical deployment of smart-home monitoring systems in aging-in-place and assisted-living applications.

A central limitation of many anomaly detection systems is that they stop at the alert. A reconstruction error, prediction error, or binary anomaly label may indicate that behavior has changed, but it does not explain the timing, location, magnitude, or possible meaning of the change. This limitation is particularly important in assisted-living contexts because not every deviation from routine represents abnormal or harmful behavior. Therefore, in this study, a routine deviation is defined as a measurable difference between expected and observed behavior for a specific resident, while abnormal behavior refers to a deviation that is clinically or contextually meaningful after expert interpretation. This distinction is important because the machine learning model can identify candidate deviation days, but human expertise is required to determine whether the deviation is benign, relevant, or potentially concerning [16,17,18].

To address these limitations, this paper proposes a two-stage framework for interpretable routine-deviation assessment in smart-home sensor networks. In the first stage, a Gated Recurrent Unit (GRU)-based routine prediction model learns personalized behavioral patterns from historical motion and contact sensor data and automatically identifies candidate deviation days based on differences between predicted and observed activity. GRU networks are suitable for this task because they can model temporal dependencies in sequential data while maintaining a relatively compact recurrent architecture [19,20,21]. In the second stage, the last seven days are visualized using radar plots against the learned routine model, allowing a human expert to visually assess deviation timing, magnitude, and behavioral meaning. This design follows a human-in-the-loop philosophy in which machine learning narrows attention to candidate days, while domain experts retain responsibility for contextual severity assessment [16,18].

The primary objective of this study is to develop and evaluate an interpretable framework for personalized routine-deviation detection in smart-home environments using unlabeled sensor data. The methodological contribution of this work is not the development of a new deep learning architecture, but the design of an integrated framework that combines participant-specific spatial-zone selection, GRU-based routine prediction, adaptive participant-specific thresholding, forecasting baseline comparison, and radar-based visual interpretation within a unified workflow. By integrating these components, the proposed framework provides an interpretable and privacy-preserving approach for identifying and explaining routine deviations while supporting human-in-the-loop behavioral assessment.

The main contributions of this work are as follows:

- An integrated framework for personalized routine-deviation detection that combines participant-specific spatial-zone selection, GRU-based forecasting, adaptive thresholding, forecasting baseline comparison, and visual behavioral interpretation.
- A preprocessing workflow that aligns heterogeneous smart-home sensor streams on a two-second master timeline and generates user-specific datasets for behavioral analysis.
- A personalized GRU forecasting model that evaluates fifteen spatial-zone combinations using five-fold time-series cross-validation to identify the most informative behavioral representation for each participant.
- An adaptive participant-specific thresholding strategy that detects candidate routine-deviation days based on daily prediction residuals.
- A radar-based visualization layer that supports post-detection interpretation of hourly behavioral patterns without contributing to the anomaly detection algorithm.
- Experimental validation using four independently living older adults, including comparisons with Previous-Day and Seasonal Naïve forecasting baselines.

## 2. Related Work

### 2.1. Smart-Home Monitoring for Aging in Place

Smart-home monitoring has been widely investigated as a non-intrusive approach for supporting aging-in-place, assisted living, and remote health monitoring. Unlike wearable or camera-based systems, ambient smart-home technologies rely on environmental sensors such as passive motion sensors, contact sensors, and door sensors to capture everyday activity patterns while preserving user privacy. Ref. [3] reviewed smart-home technologies for elderly healthcare and highlighted their potential for continuous monitoring, early risk detection, and independent living support. Ref. [4] provided a systematic review of smart-home and home health monitoring technologies for older adults, emphasizing their role in monitoring activities of daily living, chronic conditions, and functional decline. Ref. [5] also identified ambient assisted living systems as important tools for supporting older adults in residential environments.

Within smart-home environments, sensor-derived behavioral data can provide useful indicators of activity quality, health events, and changes in daily routines. Ref. [11] showed that smart environments can be used to assess the quality of activities performed by residents. Ref. [12] demonstrated that smart-home behavior data can support automated clinical assessment by linking daily activity patterns with cognitive and physical health indicators. Ref. [13] further showed that smart-home systems can detect and analyze health-related events by identifying changes in activity timing and duration. These studies demonstrate that ambient sensor data can support long-term behavioral monitoring without requiring continuous manual observation.

Routine-based interpretation is particularly important in smart-home monitoring because daily behavior is naturally organized by time, place, and personal habit. Ref. [9] showed that everyday household routines help make smart-home sensor data more understandable in aging-in-place applications. These studies suggest that personalized routine baselines are more appropriate than fixed population-level rules because each resident may express normal behavior through different activity timings, locations, and sensor-use patterns.

### 2.2. Anomaly Detection in Sensor Data

Anomaly detection in sensor networks has been studied across wireless sensor networks, Internet of Things systems, smart homes, healthcare monitoring, and industrial monitoring. Ref. [22] reviewed anomaly detection in wireless sensor networks and discussed challenges related to noise, reliability, and abnormal event identification. Ref. [23] reviewed network anomaly detection methods and highlighted the importance of detecting unusual patterns in complex data streams. Ref. [24] provided a multi-perspective review of anomaly detection in sensor systems.

Deep learning methods have become increasingly relevant for anomaly detection in time-series data because they can model nonlinear and temporal dependencies without relying on manually defined thresholds. Ref. [25] reviewed deep learning approaches for anomaly detection. Ref. [26] reviewed anomaly detection in time-series data. Recurrent neural network architectures, including Long Short-Term Memory (LSTM) and Gated Recurrent Unit (GRU) models, are particularly suitable for sequential sensor data. Ref. [19] introduced the GRU architecture as a compact recurrent model capable of learning temporal dependencies, while Ref. [20] demonstrated the use of recurrent neural networks for time-series anomaly detection.

Prediction-based anomaly detection is especially appropriate when labeled anomaly data are scarce or unavailable. Ref. [27] showed that prediction errors from recurrent models can be used for anomaly detection, while Ref. [21] applied neural networks to activity recognition, anomaly detection, and next-activity prediction in smart homes.

### 2.3. AI and Smart-Home Anomaly Detection

Recent research has advanced smart-home anomaly detection through deep learning, edge intelligence, synthetic data generation, non-wearable sensing, and personalized modeling. Ref. [14] proposed a sensor data simulation for anomaly detection among older adults living alone, addressing the lack of sufficient real anomaly data. Ref. [15] introduced a graph neural network approach for adverse health detection and personalized decision-making in sensor-based remote monitoring for dementia care. Ref. [7] proposed an IoT and edge-intelligence framework for monitoring older adults. Ref. [8] investigated the use of non-intrusive binary sensors for monitoring and detecting mobility loss in older adults. Ref. [10] developed a smart-home-assisted anomaly detection system for older adults using deep learning and a comprehensive set of daily activities.

In addition to these developments, recurrent neural network architectures have continued to play an important role in sequential prediction problems. For example, BiLSTM-based models have been successfully applied to planning and decision-making tasks, demonstrating the capability of bidirectional recurrent architectures to capture long-term temporal dependencies in dynamic environments [28]. Similarly, both LSTM and GRU networks have been widely adopted for behavioral modeling and activity prediction because of their ability to learn temporal patterns from sequential sensor observations. Compared with LSTM, however, the GRU architecture employs a simpler gating mechanism with fewer trainable parameters, resulting in lower computational complexity while maintaining comparable predictive performance in many time-series applications. Considering the relatively limited amount of participant-specific smart-home data available in this study, the GRU architecture was selected as an appropriate balance between predictive accuracy, computational efficiency, and model complexity.

Although these recent studies demonstrate strong progress, several limitations remain. In practical smart-home healthcare monitoring, anomaly detection should not only identify that a deviation occurred but also support interpretation of when it occurred, which household zone was involved, and whether the deviation appears meaningful in relation to the resident’s normal routine. This creates a need for frameworks that combine personalized routine modeling, automatic candidate anomaly detection, and interpretable visual assessment.

### 2.4. Personalized Routine Modeling in Smart Homes

Personalization is a central requirement in smart-home anomaly detection because behavioral routines differ substantially between residents. A high number of bedroom events may be normal for one person but unusual for another. Similarly, frequent door-contact activity may reflect normal outdoor mobility for one resident but a significant deviation for another. Population-level thresholds therefore risk producing false alarms when individual differences are not considered. Previous studies on smart-home behavior modeling have emphasized the need to account for resident-specific routines, activity timing, and spatial patterns. Ref. [9] showed that household routines provide a meaningful structure for interpreting sensor data.

Personalized modeling is particularly relevant when anomaly labels are unavailable. Instead of learning a universal definition of abnormal behavior, a personalized model learns what is expected for a specific resident and identifies deviations relative to that individual’s baseline. This approach is consistent with the objective of aging-in-place monitoring, where the goal is not to compare residents against a population norm but to detect meaningful changes in each person’s own behavioral pattern. In this context, routine deviation should be understood as a measurable departure from expected behavior, whereas abnormal behavior requires additional clinical or contextual interpretation.

The present study follows this personalized modeling direction by evaluating user-specific spatial zone combinations and selecting the zone that provides the most predictable routine for each resident. This strategy recognizes that different household zones may carry different behavioral importance across individuals. For some residents, bedroom activity may be the most stable routine indicator, while for others, outdoor or entrance door activity may better reflect daily structure. By selecting the most informative zone for each user, the model supports more adaptive and personalized anomaly detection.

### 2.5. Explainability and Human-in-the-Loop Assessment

Explainability is essential for smart-home anomaly detection because caregivers, clinicians, and family members must understand the context behind an alert before deciding whether action is required. A numerical anomaly score or binary label may indicate that behavior has shifted, but it fails to clarify the timing, location, magnitude, or possible meaning of the deviation. To address this limitation in black-box machine learning models, conventional methods such as Local Interpretable Model-Agnostic Explanations (LIMEs) and SHapley Additive exPlanations (SHAPs) have been widely developed [17,29]. Comprehensive reviews of explainable artificial intelligence (XAI) emphasize that limited interpretability presents both practical and ethical challenges in decision support systems, whereas recent healthcare studies highlight that transparent frameworks drastically improve clinical trust, systemic transparency, and the real-world adoption of predictive models [18].

Beyond these foundational feature importance and visualization techniques, recent developments in XAI have expanded the scope of behavioral monitoring [30]. For instance, hybrid prompt-learning approaches generate natural-language justifications for intelligent system decisions, while contextual semantic behavior graphs model complex relationships among environmental events, sensor observations, and contextual data [28]. More recently, dynamic causal graph frameworks assisted by large language models (LLMs) have been introduced to support automated root-cause analysis through graph-based causal reasoning [31]. Together, these complementary paradigms demonstrate that interpretability can be achieved through textual explanations, semantic reasoning, or explicit causal inference. In contrast to these high-overhead computational methods, the present study adopts a lightweight visual strategy. By mapping the candidate routine-deviation days identified by the GRU forecasting model onto intuitive 24-h radar plots, our framework directly supports human interpretation while maintaining absolute computational simplicity and preserving user privacy.

In healthcare and assisted-living contexts, human expertise remains necessary because sensor data alone often lack contextual information. Human-in-the-loop machine learning has been identified as particularly important when domain knowledge, contextual reasoning, and expert judgment are required [16]. Smart-home anomaly detection is a clear example of this need. A model may detect an unusual day, but a caregiver or clinician must determine whether the pattern is benign, clinically relevant, or caused by contextual factors such as visitors, weather conditions, schedule changes, holidays, maintenance activities, or sensor malfunction.

Visual analytics can help bridge the gap between automated anomaly detection and human interpretation. Instead of presenting only a prediction error or threshold-crossing event, visual representations can show how daily behavior differs from the expected routine across time and space. In the present study, seven-day radar visualization is used as an explanation layer. The radar plots compare recent daily activity against the learned routine model, allowing human experts to inspect whether deviations are concentrated at specific times of day or associated with particular household zones. This approach supports human-in-the-loop decision-making by allowing machine learning to identify candidate deviation days while leaving severity assessment and contextual interpretation to human experts.

### 2.6. Positioning of the Present Study

The reviewed literature shows that smart-home sensor networks are promising for aging in place, activity monitoring, and early detection of behavioral change. Prior studies have demonstrated the value of ambient sensing, personalized activity modeling, recurrent neural networks, and anomaly detection in smart-home environments. Recent work has further advanced the field through edge intelligence, graph neural networks, deep learning, simulated anomaly data, and binary-sensor-based mobility monitoring.

However, an important gap remains between automatic anomaly detection and practical interpretability. Many systems identify unusual behavior using prediction errors, reconstruction errors, or anomaly scores, but provide limited support for explaining the deviation in a form that can be easily interpreted by caregivers or healthcare professionals. The present study addresses this gap by proposing a two-stage framework that combines GRU-based personalized routine prediction with seven-day radar visualization. The first stage automatically identifies candidate routine-deviation days from smart-home sensor data. The second stage presents recent activity patterns against the learned routine model, enabling human experts to visually assess the timing, magnitude, and behavioral meaning of deviations. In this way, the proposed framework moves beyond black-box anomaly alerts toward interpretable, human-in-the-loop routine-deviation assessment.

Unlike recent explainable AI approaches that employ natural-language explanation generation or semantic and causal graph reasoning to interpret intelligent system decisions, the proposed framework adopts a lightweight visualization-based strategy for behavioral interpretation. Rather than introducing a new deep learning architecture, this study integrates participant-specific spatial-zone selection, GRU-based routine prediction, adaptive participant-specific thresholding, forecasting baseline comparison, and post-detection radar-based visualization within a unified workflow. Candidate routine-deviation days are identified automatically using daily prediction residuals, while the radar plots provide intuitive visual explanations of hourly behavioral patterns after detection has occurred. This approach complements existing explainable AI methods by offering an interpretable, reproducible, and privacy-preserving framework for personalized routine-deviation assessment in smart-home environments.

## 3. Material and Method

### 3.1. Smart-Home Sensor Network and Data Collection

Data collection was conducted using a smart-home monitoring system using motion sensors, door contact sensors, and environmental sensors placed in the residences of four independent older adults residing in the community. Sensor events generated by these devices provide a non-intrusive representation of daily activities and interactions with household spaces and objects. Each participant was monitored continuously for 28 consecutive days.

**Figure 1:**
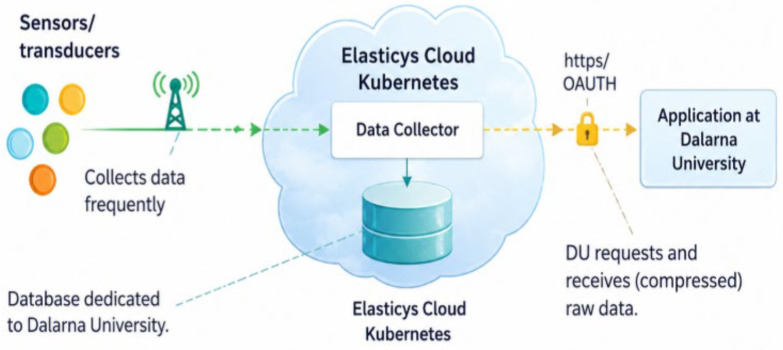
Smart-home monitoring infrastructure.

Figure 1 presents the smart-home monitoring infrastructure used in this study. Sensor events were transmitted from the residential sensing network to a cloud-based storage environment, where the data were securely maintained and subsequently accessed for behavioral analysis, machine learning model development, routine prediction, and deviation detection.

### 3.2. Sensor Placement and Behavioral Monitoring

**Figure 2:**
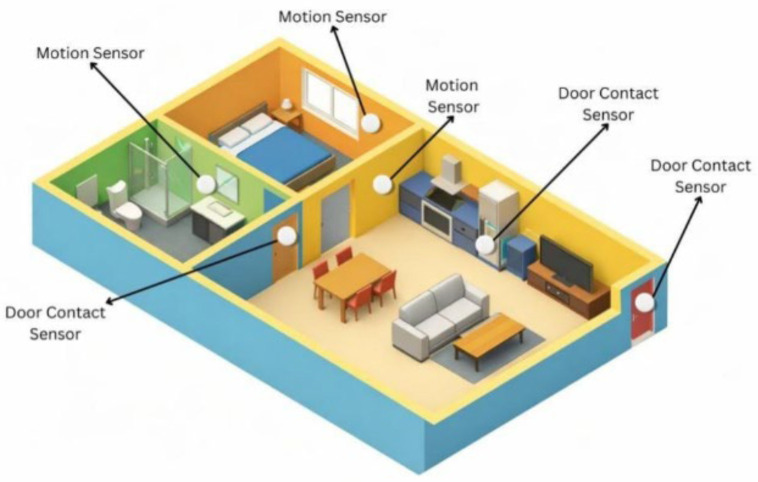
Sensor placement layout.

The monitored houses had both types of detectors: motion sensors and contact sensors. Motion sensors were set up in important parts of the house where activity took place, whereas contact sensors detected interaction between people and objects as well as access. Figure 2 shows the location of the motion and contact sensors in the monitored smart home.

To make the raw sensor streams behaviorally meaningful, individual sensor-event columns are mapped into four household zones (Table 1). Kitchen/Living combines the first motion sensor with the second door-contact open and close events. Outdoor combines the first door-contact open and close events. Bedroom uses the second motion sensor. Washroom combines the third motion sensor with the third door-contact open and close events.

### 3.3. Dataset Characteristics

This dataset is made up of motion and contact sensor events recorded for four separate smart home users (User 1 to User 4) over 28 days. Table 2 summarizes the characteristics of the collected motion sensor dataset for all participants. Considerable variability can be observed across users in terms of total event counts, event density, and occupancy distributions. Users 2 and 4 generated substantially higher numbers of motion sensor events (40,242 and 41,366 events, respectively) than Users 1 and 3, indicating more active behavioral patterns throughout the monitoring period.

In order to test the proposed approach with varied behavior types, selection of subjects was done based on the availability of complete data for a full monitoring period, along with adequate sensor activities. Inclusion of various subjects gave the advantage of studying the proposed approach across varied activity levels of households.

Table 3 presents summary statistics for contact sensor activity. Contact sensor events complement motion sensor observations by capturing interactions with household objects and access points.

### 3.4. Data Preprocessing

Raw sensor streams contain inconsistencies such as duplicated events, timestamp irregularities, and heterogeneous sensor frequencies. Therefore, a comprehensive preprocessing pipeline was developed before model training.

First, timestamp synchronization was performed to ensure temporal consistency across all sensor streams. Motion and contact sensor events were chronologically ordered and aligned to a common temporal reference. Duplicate records were removed, and missing values were handled where necessary. Subsequently, temporal features including date, hour of day, and day of week were extracted from sensor timestamps.

Because sensor activations do not necessarily occur during every hour of the day, some hourly intervals contained no recorded events. To maintain continuous temporal sequences required for recurrent neural network training, missing hourly observations were handled using a forward-filling strategy. This approach propagates the most recent activity state to subsequent empty intervals, ensuring complete temporal coverage while preserving behavioral continuity. The resulting continuous time-series representation improves model stability and facilitates the learning of daily behavioral patterns.

To reduce noise and improve interpretability, sensor events were subsequently aggregated into hourly activity counts. Hourly aggregation provides an effective balance between temporal resolution and computational efficiency for smart-home activity modeling. For the 28-day monitoring period, hourly aggregation produced 672 hourly observations per participant. Following preprocessing and aggregation, all sensor events were transformed into structured hourly behavioral profiles suitable for sequence learning and predictive modeling.

### 3.5. Spatial-Zone Construction

Individual sensor events were grouped into four functional household zones: Kitchen/Living, Bedroom, Washroom, and Outdoor/Entrance areas. This spatial grouping transformed low-level sensor activations into semantically meaningful activity representations that better reflect daily behavioral routines.

The Kitchen/Living zone captured meal preparation and general living activities through movement and Kitchen-related interactions. The Bedroom zone represented rest-related presence and movement. The Washroom zone reflected hygiene-related activities through bathroom motion and access events. The Outdoor zone captured entry and exit behavior through interactions with the main entrance door.

To investigate how spatial information influences routine prediction performance and candidate deviation identification, all possible non-empty combinations of the four zones were generated. This resulted in fifteen unique spatial configurations. Each configuration was independently evaluated during model development, enabling data-driven selection of the most informative behavioral representation for each participant.

### 3.6. Routine Prediction Using GRU Networks

Behavioral routine prediction was performed using a Gated Recurrent Unit (GRU) neural network. GRU networks are well suited for smart-home activity modeling because they effectively capture temporal dependencies in sequential sensor data while requiring fewer trainable parameters than conventional Long Short-Term Memory (LSTM) networks, resulting in improved computational efficiency and reduced training complexity.

The selection of GRU was motivated by both methodological and practical considerations. Smart-home monitoring datasets are often relatively small and contain limited labeled examples of clinically or contextually validated deviation events. Compared with LSTM architectures, GRU networks employ a simpler gating mechanism with fewer trainable parameters while retaining the ability to model temporal dependencies effectively. This reduced architectural complexity lowers the risk of overfitting, improves computational efficiency, and enables efficient learning from relatively small participant-specific datasets. Consequently, the GRU architecture was selected as an appropriate balance between predictive accuracy, computational efficiency, and model complexity for personalized smart-home behavioral modeling. The objective of the GRU model was to learn normal behavioral routines and generate predictions of future activity levels based on historical sensor observations. Daily activity sequences derived from the selected spatial zones were used as model inputs with a three-day look-back window to capture short-term temporal dependencies and recurring behavioral patterns.

Prior to model training, activity counts were normalized using Min–Max scaling to ensure numerical stability and facilitate efficient optimization. To prevent data leakage, normalization parameters were estimated exclusively from the training data and subsequently applied to validation and testing observations.

**Figure 3:**
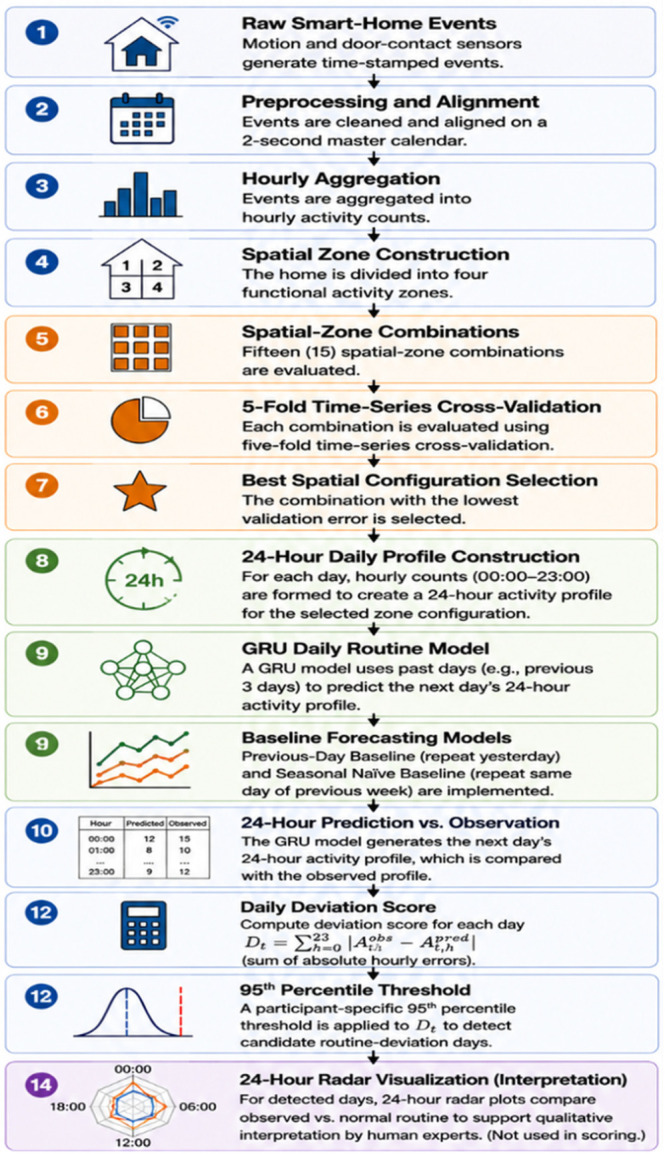
Workflow of the proposed GRU-based prediction framework with baseline comparison for routine-deviation detection in a smart-home environment.

The implemented network architecture consisted of a GRU layer containing 32 hidden units, followed by a dense hidden layer with 16 ReLU neurons and a linear output layer responsible for generating activity predictions. Model training was performed using the Adam optimization algorithm [32] with mean squared error as the loss function. Early stopping was employed to reduce overfitting, with a maximum of 100 training epochs and a batch size of four. The best-performing model weights were automatically restored based on validation loss. This configuration enabled the network to learn participant-specific behavioral dynamics while maintaining a compact and computationally efficient architecture. Figure 3 illustrates a workflow of the proposed GRU-based routine-deviation detection framework. Raw smart-home sensor events are preprocessed and aggregated into hourly activity counts. Four functional spatial zones are constructed, and fifteen spatial-zone combinations are evaluated using five-fold time-series for cross-validation. The best-performing spatial configuration is selected to train the proposed GRU forecasting model, while Previous-Day and Seasonal Naïve predictors are included as baseline models for performance comparison. Daily routine deviation is computed as the absolute difference between predicted and observed daily activity counts. Candidate routine-deviation days are identified using a participant-specific 95th percentile threshold, and 24-h radar plots are generated solely for qualitative interpretation of the detected routine deviations.

Model performance was assessed using Root Mean Square Error (RMSE) and Mean Absolute Error (MAE) [33]. To evaluate the benefit of the recurrent forecasting model, the proposed GRU was compared with two simple forecasting baselines: a Previous-Day predictor and a Seasonal Naïve predictor. Both baselines were evaluated using the same five-fold time-series cross-validation protocol.

### 3.7. Five-Fold Time-Series Cross-Validation

To ensure robust model evaluation and avoid temporal leakage, a five-fold time-series cross-validation strategy was adopted. Unlike traditional random cross-validation approaches, time-series cross-validation preserves the chronological ordering of observations by using earlier observations for training and later observations for validation. For each participant, all fifteen spatial zone configurations were independently evaluated using the GRU prediction framework.

**Figure 4:**
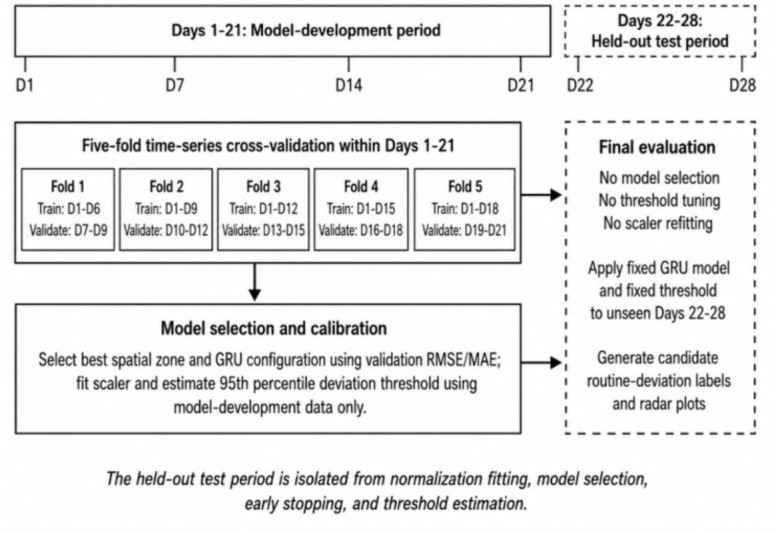
Chronological data partitioning and evaluation protocol.

The chronological data partitioning strategy is illustrated in Figure 4. For each participant, the 28-day monitoring period was divided into a model-development period and a held-out test period. Days 1–21 were used for model development, including Min–Max scaler fitting, five-fold time-series cross-validation, spatial-zone selection, model selection, and threshold calibration. Days 22–28 were retained as a chronologically held-out test period and were not used during normalization fitting, model selection, early stopping, spatial-zone selection, or threshold estimation. This separation ensured that the final evaluation was performed only on previously unseen observations.

Within the 21-day model-development period, five-fold time-series cross-validation was performed using expanding chronological windows. In each fold, the GRU model was trained on earlier days and validated on later days. The validation results were used to select the best participant-specific spatial-zone configuration according to RMSE, with MAE used as a secondary metric. After the optimal configuration was selected, the final GRU model was retrained using the full model development period and then applied once to the held-out test period.

To evaluate the predictive advantage of the proposed GRU model over simple deterministic forecasting methods, two standard forecasting baselines were implemented. The first baseline was a Previous-Day baseline (Naïve Predictor), which forecasts the activity count for the current day using the observations from the immediately preceding day. The second baseline was a Seasonal Naïve baseline, which forecasts the activity count using the observations from the corresponding day of the previous week. Both baseline models were evaluated using the same five-fold time-series cross-validation protocol and the same evaluation metrics (RMSE and MAE) as the proposed GRU model, thereby providing a fair comparison of forecasting performance.

### 3.8. Deviation Analysis and Temporal Routine Visualization

Following the identification of the optimal participant-specific spatial-zone configuration, the full 21-day model development window (days 1–21) was utilized to train the final GRU network and calibrate the baseline threshold. The chronologically isolated held-out period (days 22–28) was then used as the testing phase to identify candidate routine deviations. To prevent data leakage and guarantee experimental reliability, these final seven testing days remained completely separate from model architecture selection, early stopping triggers, spatial-zone evaluation, and normalization scale fitting.

For each operational test day within this evaluation window, the activity profile forecasted by the trained GRU model was compared against the actual observed activity count. To quantify the exact magnitude of the discrepancy, a daily deviation metric was computed as Equation (1):

(1) $$D_{t}=\left|A_{t}-\hat{A_{t}}\right|$$

where *D_t_* denotes the daily deviation score at day *t*, *A_t_* represents the actual observed daily activity count, and $\hat{A_{t}}$ signifies the activity count forecasted by the GRU model.

To identify unusually large deviations from normal day-to-day behavioral variation, an adaptive, percentile-based thresholding approach was employed. The candidate-deviation threshold (*τ*) was calculated as Equation (2):

(2) $$\tau =P_{95}(D_{train})$$

where *P*_95_ represents the 95th percentile of the empirical distribution of deviation scores observed during the training period. Days with deviation scores greater than or equal to the participant-specific threshold were classified as candidate routine-deviation days. The proposed routine-deviation detection algorithm operates exclusively at the daily level, where each test day is represented by a single prediction residual computed using Equation (1). Therefore, the anomaly score is derived solely from daily activity counts rather than hourly behavioral profiles, as defined in Equation (3):

(3) $$Y_{t}=\left\{\begin{array}{c} 1,\;if\;D_{t}\geq \tau \\ 0,\;if\;D_{t}<\tau \end{array}\right.$$

where *Y_t_* = 1 indicates a candidate routine-deviation day that warrants contextual human-in-the-loop review, and *Y_t_* = 0 represents behavior consistent with the learned routine baseline. After candidate routine-deviation days have been identified, 24-h radar plots are generated to facilitate qualitative interpretation of behavioral changes by comparing the hourly activity profile of the detected day with the participant-specific normal routine. These radar plots are not used to calculate the anomaly score or determine whether a day is classified as a routine deviation; instead, they serve solely as an explanatory visualization that helps interpret the timing, location, and magnitude of the detected behavioral changes.

## 4. Results

### 4.1. Spatial-Zone Selection

To identify the most informative behavioral representation for each participant, fifteen spatial-zone combinations derived from the four activity zones (Kitchen/Living, Outdoor, Bedroom, and Washroom) were evaluated using five-fold time-series cross-validation. The GRU model was trained and validated separately for each combination, and prediction accuracy was assessed using RMSE and MAE metrics (Table 4). The results indicate that the optimal behavioral representation differed across participants. Users 1 and 3 achieved the lowest prediction errors when Bedroom activity was used as the forecasting target, whereas Outdoor activity yielded the best performance for Users 2 and 4. The obtained RMSE values ranged between 0.136 and 0.180. A notable observation is that single-zone configurations consistently outperformed multi-zone combinations.

Table 4 compares the forecasting performance of the proposed GRU model with the Previous-Day and Seasonal Naïve forecasting baselines. The proposed GRU consistently achieved lower RMSE and MAE values than the Previous-Day baseline for all four participants, demonstrating its ability to capture temporal dependencies beyond simple persistence. Furthermore, the GRU outperformed the Seasonal Naïve Baseline for Users 1, 2, and 4. For User 3, the Seasonal Naïve baseline achieved a slightly lower RMSE (0.161 versus 0.170), whereas the proposed GRU achieved the lowest MAE (0.138 versus 0.145). This result suggests that User 3 exhibited a highly regular weekly behavioral routine that could be effectively modeled using seasonal persistence. Overall, the baseline comparison demonstrates that the proposed GRU generally provides superior forecasting performance while effectively modeling participant-specific temporal dependencies beyond those captured by simple persistence-based forecasting methods.

**Figure 5:**
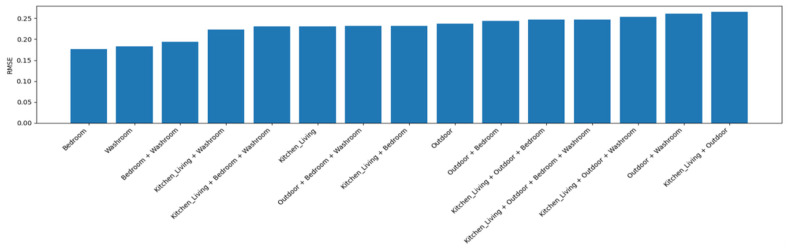
GRU prediction performance across spatial-zone combinations for User 1.

**Figure 6:**
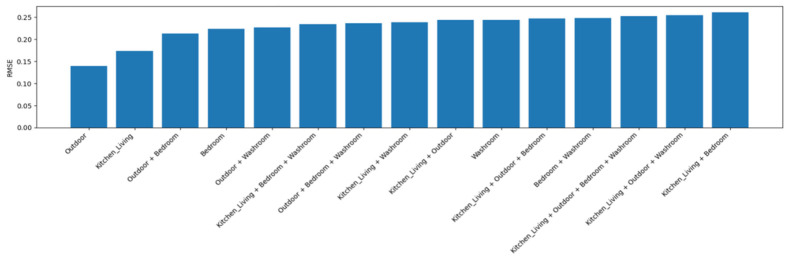
GRU prediction performance across spatial-zone combinations for User 2.

**Figure 7:**
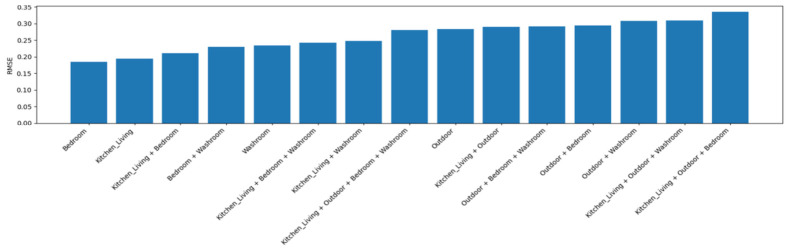
GRU prediction performance across spatial-zone combinations for User 3.

**Figure 8:**
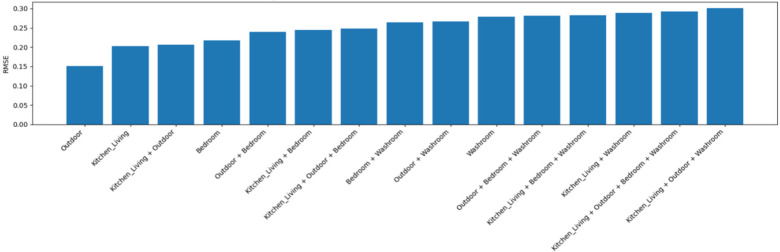
GRU prediction performance across spatial-zone combinations for User 4.

The performance distributions shown in Figure 5, Figure 6, Figure 7 and Figure 8 further confirm that prediction accuracy was highly dependent on the selected behavioral zone. For User 1, the Bedroom zone produced the lowest RMSE and MAE values, indicating that bedroom-related activities followed a highly regular temporal structure. A similar pattern was observed for User 3. In contrast, Users 2 and 4 exhibited more predictable Outdoor activity patterns, suggesting that entrance door usage and mobility-related behavior represented the dominant routine characteristics for these participants. These findings demonstrate that behavioral routines are highly individualized and support the use of personalized spatial representations for smart-home monitoring systems.

### 4.2. Hourly Behavioral Pattern Analysis

The proposed framework detects candidate routine-deviation days using the daily deviation score defined in Equation (1). Consequently, the radar plots presented in this section should be interpreted as post-detection visualization tools rather than as part of the anomaly detection algorithm. Their purpose is to assist in understanding how the hourly distribution of activities differs from the participant-specific normal routine after a routine deviation has been detected.

To further investigate the temporal characteristics of candidate routine-deviation days, hourly activity profiles were compared against the learned routine baseline for each participant. These radar plots provide an intuitive representation of how activity distributions varied across the 24-h day and facilitate qualitative interpretation of detected behavioral changes.

**Figure 9:**
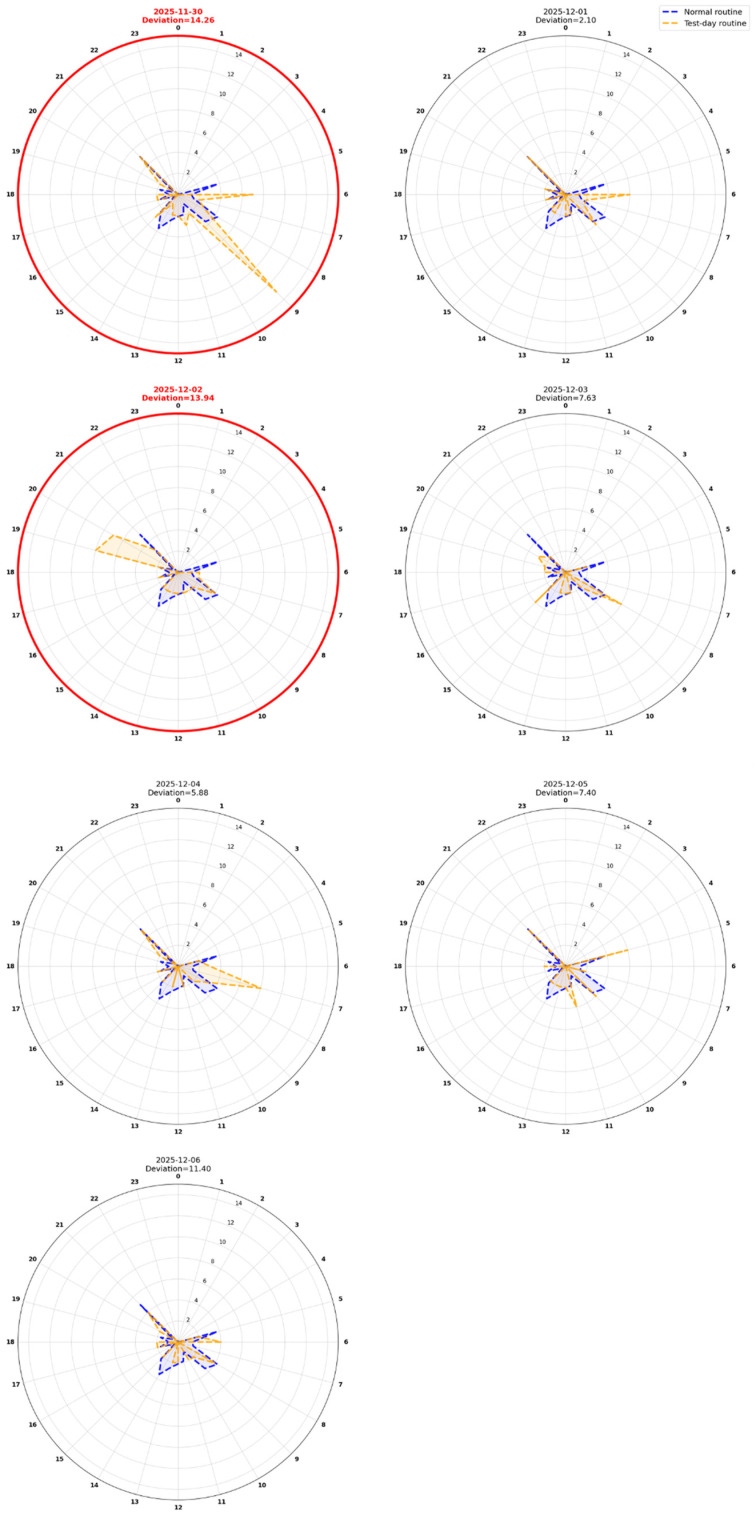
The 24-h bedroom routine for User 1. Model routine in blue and test days in orange.

User 1—As shown in Figure 9, the Bedroom activity profile for User 1 remained relatively stable, except for the two days with the highest deviation scores (30 November and 2 December), which exceeded the 95th percentile candidate-deviation threshold (red radars on Figure 9) and exhibited noticeable departures from the baseline profile, particularly during morning and evening hours. Additionally, 6 December ranked among days with the highest deviation and showed a visibly altered activity distribution in the radar plot, although its deviation score remained below the formal candidate-deviation threshold. These deviations suggest temporary changes in the participant’s usual bedroom-related routine, although their underlying cause cannot be determined from the available sensor data alone.

**Figure 10:**
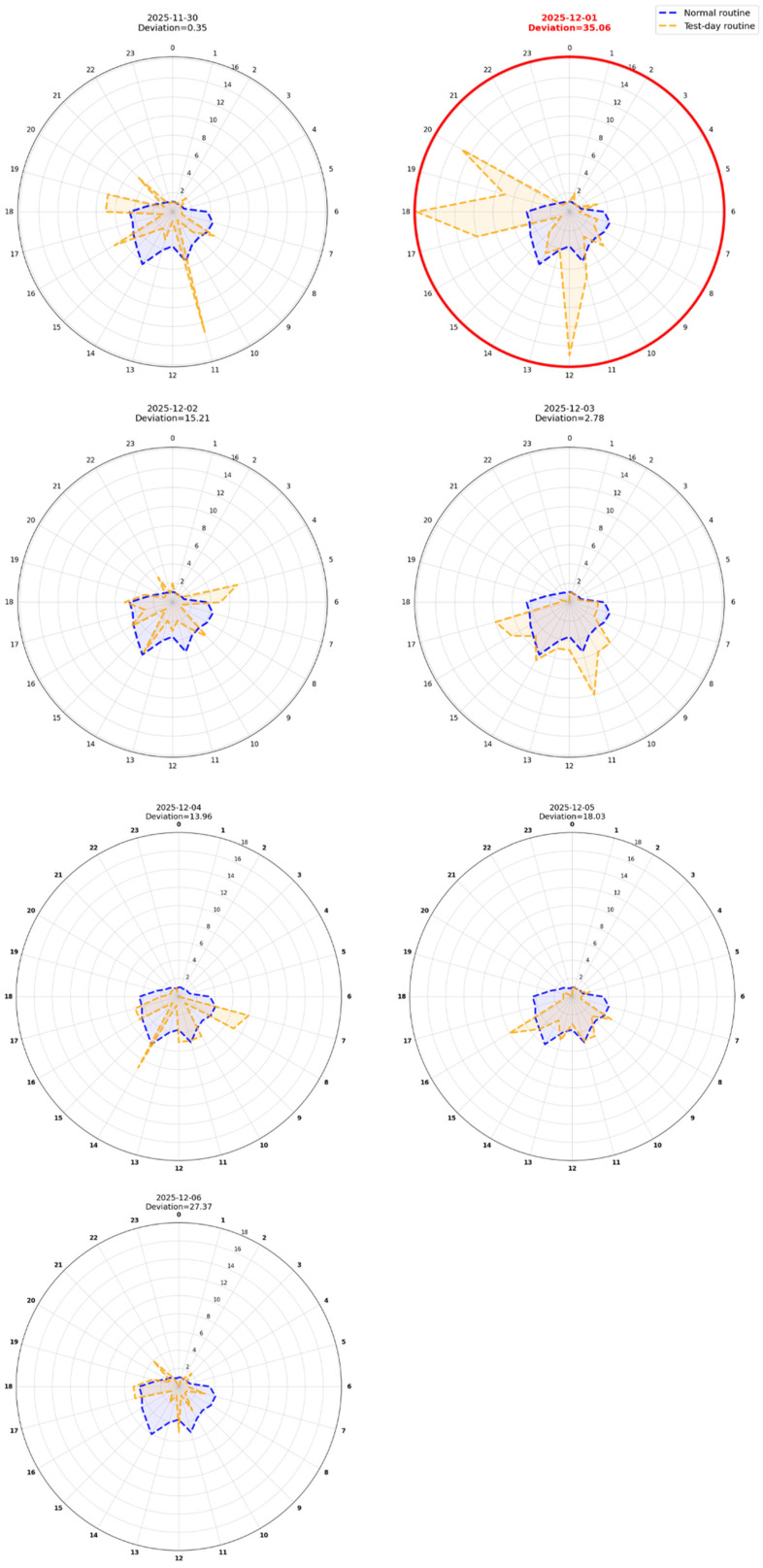
The 24-h outdoor routine for User 2. Model routine in blue and test days in orange.

User 2—Figure 10 illustrates outdoor activity patterns for User 2. The difference between the routine model and actual activity is observed on 1 December, which exceeded the candidate-deviation threshold (red radar on Figure 10). With several hourly activity counts substantially exceeding the baseline pattern, this observation corresponds to the largest single deviation observed across all participants. These findings suggest changes in mobility-related behavior and entrance door usage during the candidate deviation periods, although clinical or contextual interpretation would require additional information.

**Figure 11:**
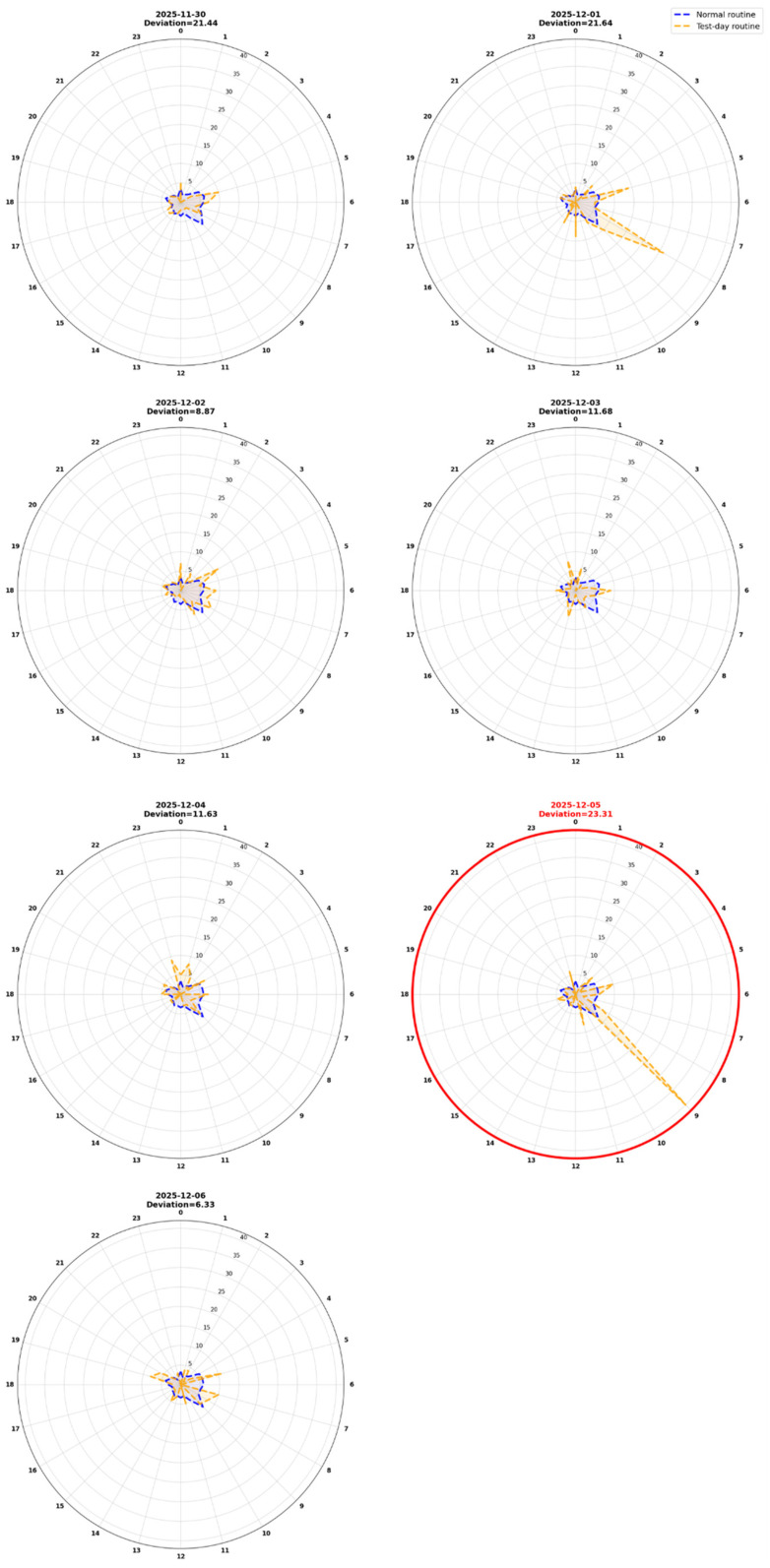
The 24-h bedroom routine for User 3. Model routine in blue and test days in orange.

User 3—Figure 11 shows that Bedroom activity generally followed a consistent routine profile. Moreover, 5 December exceeded the anomaly threshold (red radar on Figure 11) and exhibited a localized activity spike and shift in activity timing relative to the learned baseline. Additionally, 30 November and 1 December also ranked among days with the most deviations and showed comparable shifts in timing but did not exceed the formal threshold. These results indicate that the highest candidate deviation occurred on 5 December, with related but less pronounced variations occurring earlier in the test period.

**Figure 12:**
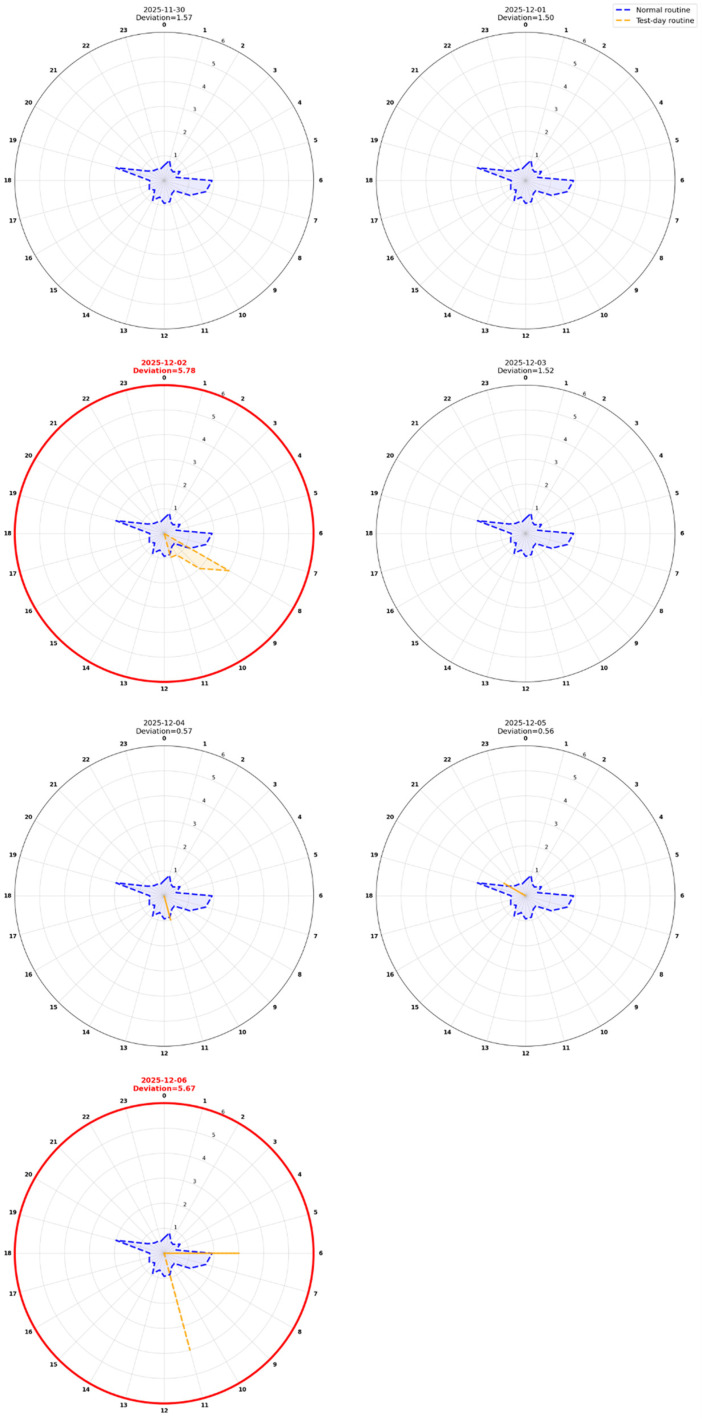
The 24-h outdoor routine for User 4. Model routine in blue and test days in orange.

User 4—As illustrated in Figure 12, User 4 exhibited lower overall Outdoor activity levels than the other participants. Despite this lower activity volume, candidate routine-deviation days (2 December and 6 December) exceeded the candidate-deviation threshold (red radars on Figure 12) and displayed clear departures from the normal routine profile. This suggests that the proposed framework can identify large deviations from a participant-specific routine baseline even within low-activity environments.

Overall, the radar visualizations suggest that the highest-deviation days were associated with changes in the timing and distribution of activities throughout the day rather than only increases or decreases in total activity counts. These findings complement the quantitative deviation score results and provide interpretable visual information about how candidate routine-deviation days differed from the learned participant-specific baseline.

### 4.3. Candidate Routine Deviation

Following the identification of the optimal spatial zone configuration for each participant, the GRU model was retrained using the complete training dataset and subsequently evaluated on previously unseen test days. Predicted daily activity counts were compared with observed sensor events to assess the model’s ability to learn participant-specific behavioral routines and identify candidate deviations from those routines.

**Figure 13:**
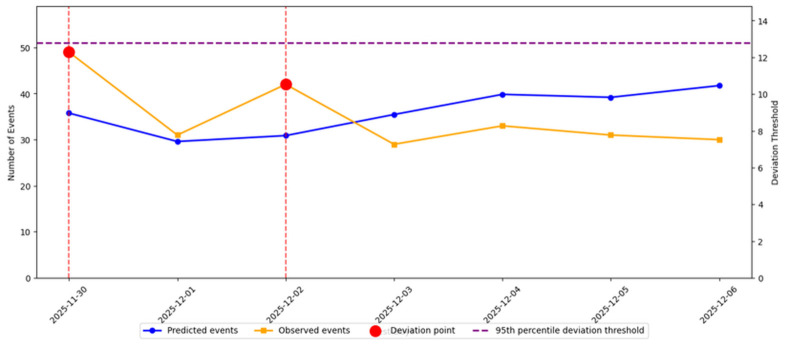
Predicted versus observed bedroom activity for User 1. The red dotted lines indicate the deviation days.

**Figure 14:**
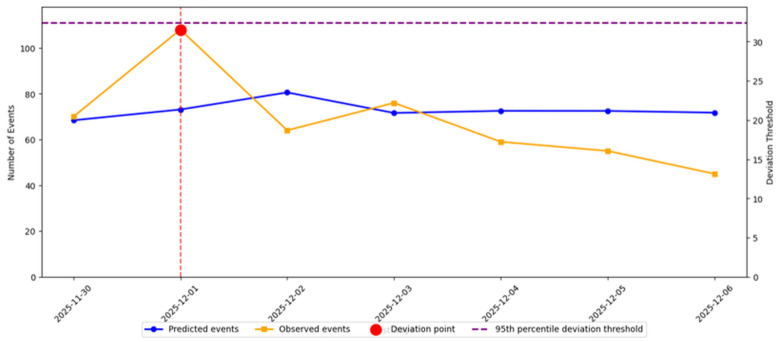
Predicted versus observed outdoor activity for User 2. The red dotted line indicates a deviation day.

**Figure 15:**
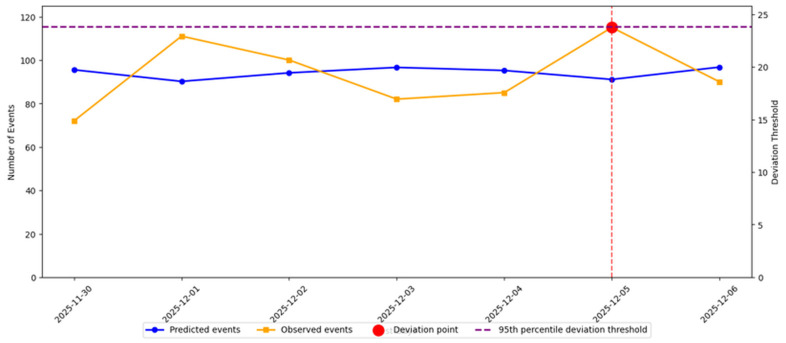
Predicted versus observed bedroom activity for User 3. The red dotted line indicates a deviation day.

**Figure 16:**
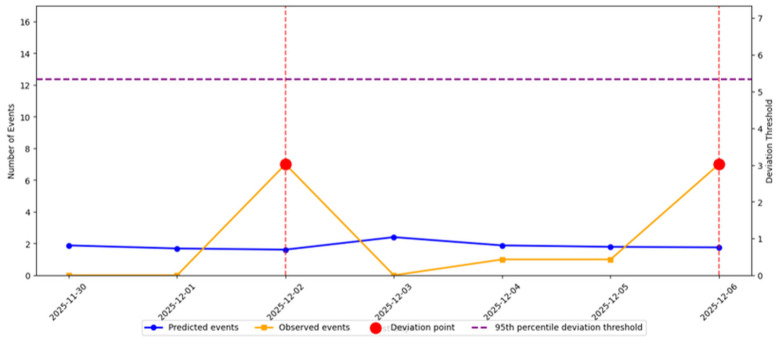
Predicted versus observed outdoor activity for User 4. The red dotted lines indicate the deviation days.

Figure 13, Figure 14, Figure 15 and Figure 16 present the predicted and observed activity counts for each participant together with the participant-specific candidate-deviation threshold derived from the 95th percentile of the training deviation distribution. Days exceeding this threshold were labeled as candidate routine-deviation days. For most test days, the predicted activity counts closely followed the observed activity levels, indicating that the GRU model successfully captured recurring behavioral patterns and established reliable individualized baselines.

User 1—As shown in Figure 13, the GRU model is relatively close to the normal Bedroom activity pattern for User 1 across most test days. However, substantial deviations were observed on 30 November and 2 December, where observed activity levels differed considerably from the predicted baseline and exceeded the candidate-deviation threshold. These deviations suggest temporary changes in the participant’s usual bedroom routine, although the available data do not allow the underlying cause to be determined.

User 2—Figure 14 shows the difference between the predicted and actual Outdoor activity for User 2. Additionally, 1 December produced a deviation score exceeding the anomaly threshold, with observed activity substantially exceeding the predicted level, indicating a marked, isolated change in mobility-related behavior.

User 3—Figure 15 shows that the predicted Bedroom activity closely matches the observed activity during the seven-day test period. However, one day (5 December) exhibited a substantial discrepancy between predicted and observed activity counts, resulting in elevated deviation scores and candidate anomaly detection.

User 4—As illustrated in Figure 16, User 4 displayed comparatively stable Outdoor activity throughout the testing period. Although overall activity levels remained low, candidate deviations were identified on 2 December and 6 December, suggesting that the proposed framework can detect large departures from a participant-specific baseline even in environments characterized by sparse sensor activity.

The close agreement between predicted and observed activity counts on routine-consistent days suggests that the GRU model learned individualized behavioral patterns in this proof-of-concept dataset. Importantly, the detected candidate deviation days should not be interpreted as evidence of abnormal, unhealthy, or clinically meaningful behavior. Rather, they represent unusually large departures from each participant’s learned routine, as determined by the predictive baseline established by the GRU model.

Furthermore, the identified candidate deviations reflected both changes in overall activity volume and shifts in the temporal distribution of activities throughout the day. The participant-specific thresholding strategy allowed deviation identification to be adapted to individual behavioral variability. By defining candidate deviations relative to each participant’s learned routine rather than using a fixed global threshold, the framework aimed to reduce detections caused only by natural differences in activity levels across individuals.

## 5. Discussion

### 5.1. Personalized Spatial-Zone Selection

One of the most significant findings of this study is that the optimal behavioral representation varied across participants. Users 1 and 3 achieved the lowest prediction errors when Bedroom activity was modeled, whereas Outdoor activity provided the strongest predictive signal for Users 2 and 4. This observation suggests that behavioral routines may be highly individualized and that different residents may express their daily habits through different household locations.

The cross-validation results further revealed that single-zone models consistently outperformed multi-zone combinations. Although combining multiple spatial zones increases the amount of available behavioral information, it also introduces additional variability and noise that may reduce forecasting accuracy. In the present study, the Bedroom and Outdoor zones captured the most stable and repetitive activity patterns, resulting in lower RMSE and MAE values than more complex spatial representations.

These findings are consistent with previous research emphasizing the importance of personalized activity modeling in smart-home environments. Rather than relying on a universal representation of behavior, anomaly detection systems may benefit from identifying the most informative behavioral context for each resident [12,34].

### 5.2. Effectiveness of GRU-Based Routine Prediction

The GRU prediction framework was able to model participant-specific behavioral routines and generate activity forecasts during routine-consistent periods [19]. Across all four participants, predicted activity counts closely followed observed activity patterns for the majority of testing days, suggesting that the model captured recurring temporal dependencies within the sensor data.

The relatively low RMSE and MAE values obtained during five-fold cross-validation suggest that routine activity patterns were sufficiently stable to be modeled using short-term temporal dependencies. The three-day look-back window appeared adequate for capturing recurring behavioral cycles while maintaining model simplicity and computational efficiency.

An important observation is that prediction errors increased substantially only during specific test days. This behavior indicates that the model was not simply memorizing activity levels but was learning underlying routine structures. Consequently, large prediction errors were treated as indicators of possible routine disruption rather than as direct evidence of clinically meaningful events.

An additional advantage of the proposed prediction-based approach is its suitability for unlabeled behavioral datasets. Obtaining clinically validated anomaly labels from older adults is often costly, time-consuming, and ethically challenging. By learning normal routine patterns directly from historical observations, the GRU framework can identify candidate routine deviations without requiring extensive manual annotation. This characteristic supports the potential applicability of the framework for future smart-home monitoring studies [20,25].

### 5.3. Behavioral Interpretation of Candidate Routine Deviations

The proposed framework identified candidate routine-deviation days characterized by large departures from learned participant-specific routines. Importantly, these candidate deviation days should be interpreted as statistical deviations from each participant’s established routine rather than confirmed abnormal, unhealthy, or clinically meaningful events. The proposed framework identifies days on which observed activity patterns differ substantially from the learned behavioral baseline; however, it cannot determine the underlying cause of these deviations. A routine deviation may result from normal lifestyle changes, altered schedules, social visits, environmental factors, temporary illness, sensor-related issues, or other contextual influences. Therefore, detected candidate deviations should be regarded as behavioral changes that may warrant further contextual review rather than definitive indicators of health-related problems.

For Users 1 and 3, candidate deviations were primarily associated with Bedroom activity. Radar visualizations revealed periods of unusually high bedroom activity and temporal shifts in routine occupancy patterns. Such changes may be associated with altered sleep schedules, extended resting periods, temporary illness, working from home, late-night activity, or other lifestyle-related factors [12,13].

For Users 2 and 4, candidate deviations were primarily observed within Outdoor activity patterns. Elevated Outdoor activity may indicate increased mobility, social visits, maintenance activities, or outdoor tasks, whereas reduced Outdoor activity may reflect prolonged indoor stay, environmental influences, schedule changes, or temporary reductions in mobility.

It is worth noting that the days with the highest relative deviation scores did not always exceed the absolute anomaly threshold; for example, 6 December for User 1 and 5–6 December for User 2 ranked among the most deviant days yet did not exceed the formal candidate-deviation threshold. From a caregiving perspective, this distinction is meaningful as a system that surfaces only threshold-crossing alerts may overlook days that, while not extreme outliers, still represent a noticeable departure from a resident’s established routine, such as a later bedtime, a skipped outing, or a quieter morning than usual. Rather than discarding sub-threshold deviations as noise, future implementations might present them as lower-priority “soft signals” to caregivers, who are often best positioned to interpret whether a subtle change is meaningful given the context the system cannot observe (a planned family visit, a reported minor illness, a change in weather). A layered review approach, with higher-priority review for threshold-crossing days and lower-priority review for high-ranking but sub-threshold days, could make such a system feel less like a binary alarm and more like a familiar companion that notices small changes the way an attentive caregiver would.

The hourly routine comparisons further suggested that candidate deviations were often concentrated within specific periods of the day rather than being distributed uniformly across the entire 24-h cycle. This finding suggests that candidate routine deviations may involve temporal shifts in activity timing rather than only increases or decreases in total activity volume.

An important consideration is that routine deviations may originate from multiple sources beyond behavioral change itself. Sensor failures, temporary communication interruptions, environmental conditions, visitor activity, household maintenance, holidays, and other contextual factors may also contribute to deviations detected by the framework. Consequently, detected candidate deviations should be interpreted as indicators of possible routine disruption that warrant contextual review rather than definitive evidence of a particular behavioral or clinical event.

Alternative explanations may include visitor presence, changes in household schedules, temporary sensor malfunction, environmental conditions, seasonal activities, maintenance work, or social engagements. Human interpretation and contextual information therefore remain essential for determining the significance of detected deviations [16,24].

### 5.4. Contribution of Visual Behavioral Analytics

A key contribution of the proposed framework is the integration of candidate routine-deviation identification with interpretable visual analytics. While deviation scores provide a quantitative measure of departure from the learned routine baseline, they do not explain how a candidate deviation day differs from routine-consistent behavior.

The radar visualizations revealed temporal characteristics that were not immediately apparent from daily activity counts alone. In several cases, candidate deviation days exhibited activity peaks concentrated within specific hourly intervals, whereas routine-consistent days displayed more evenly distributed activity patterns. These visual differences provided additional insight into the nature of the detected candidate deviations and facilitated interpretation of the deviation-score results.

Such visual representations may be useful in future healthcare and assisted-living research contexts where clinicians and caregivers require understandable explanations for candidate deviation alerts [15]. The ability to combine predictive analytics with interpretable behavioral visualizations may improve transparency and practical usability, although user-centered validation is required to assess its impact on trust and decision-making [16].

### 5.5. Implications for Smart-Home Healthcare Monitoring

The results suggest the potential of personalized behavioral monitoring for supporting independent living and future decision support research. By learning resident-specific routines, the proposed framework can identify candidate routine deviations that may warrant contextual review.

Changes in activity patterns have previously been associated with cognitive decline, mobility impairment, sleep disturbances, and other health-related conditions. Although the present study does not establish causal relationships between candidate deviations and clinical outcomes, the ability to identify deviations from learned routines provides a basis for future health-monitoring studies with contextual or clinical validation.

Because the framework relies exclusively on non-intrusive motion and contact sensors, it offers an attractive alternative to camera-based monitoring systems. The preservation of privacy, combined with continuous behavioral assessment, suggests that the approach may be suitable for further investigation in long-term residential monitoring studies.

An important methodological distinction of the proposed framework is the separation between automated routine-deviation detection and visual interpretation. Candidate routine-deviation days are identified exclusively using the daily prediction residual defined in Equation (1), whereas the 24-h radar plots are generated only after detection to facilitate qualitative interpretation of hourly behavioral patterns. Consequently, the proposed framework should be regarded as a two-stage decision support approach in which the GRU forecasting model automatically identifies candidate routine-deviation days, while the radar visualizations assist researchers or healthcare professionals in interpreting the temporal characteristics of the detected behavioral changes.

### 5.6. Limitations

The first limitation of this study is the dataset size, which consists of only four independently living older adults monitored over a period of 28 consecutive days. Although the proposed framework demonstrated promising performance across all participants, the present study should be regarded as a proof-of-concept evaluation rather than evidence of statistical generalizability. The relatively small cohort limits the ability to assess inter-individual variability, demographic effects, seasonal changes, and rare behavioral events. Future studies involving larger and more diverse participant cohorts, longer monitoring periods, and multiple residential environments are required to further validate the robustness and applicability of the proposed framework.

The second limitation is the fact that the candidate routine-deviation identification was performed using deviations between predicted and observed activity patterns rather than independently validated behavioral or clinical events. Consequently, the detected candidate deviation days should be interpreted as statistically unusual routine deviations rather than confirmed health-related anomalies. Additional contextual or clinical information would be required to determine the underlying cause and significance of the detected behavioral changes.

Third, the study relied exclusively on passive motion sensors and contact sensors. While these sensors provide privacy-preserving behavioral information, they represent indirect indicators of human activity. For example, elevated Outdoor activity inferred from door-contact events may reflect increased mobility, visitor interactions, household maintenance activities, or other contextual factors. Similarly, increased Bedroom activity may result from altered sleep schedules, temporary illness, work-from-home routines, or unrelated lifestyle changes.

Fourth, the spatial zone representation was constructed using predefined sensor groupings and does not directly infer semantic activities such as cooking, sleeping, bathing, or social interaction. Consequently, the framework identifies deviations in activity patterns rather than deviations in specific daily living activities.

Fifth, although the proposed framework demonstrated promising predictive performance, computational efficiency and deployment feasibility were not evaluated systematically. Specifically, metrics such as training time, inference time, memory consumption, and execution on resource-constrained edge devices were beyond the scope of the present study. Since the primary objective of this work was to evaluate the effectiveness of personalized routine-deviation detection, computational benchmarking was not performed. Future work will investigate these aspects to assess the suitability of the proposed framework for real-time smart-home monitoring and practical healthcare deployment.

Another important limitation relates to the absence of ground-truth anomaly labels. The dataset contains naturally occurring behavioral observations rather than clinically or contextually verified events. Consequently, candidate routine-deviation identification was evaluated using prediction errors, deviation scores, and behavioral visualization rather than direct comparison with confirmed health outcomes. Therefore, standard anomaly detection metrics such as precision, recall, F1-score, and AUC could not be meaningfully computed in the present study. Furthermore, the participant-specific 95th percentile threshold used to distinguish routine-deviation days from normal behavioral variation represents an empirical choice and therefore introduces a degree of subjectivity into the detection process. While these limitations are common in smart-home monitoring research, future studies incorporating contextual logs, caregiver reports, clinically validated annotations, and data-driven threshold optimization will enable more objective evaluation using standard anomaly detection metrics.

Finally, environmental and contextual variables such as weather conditions, medication schedules, caregiver observations, calendar events, and social interactions were not available within the dataset. Incorporating such contextual information could improve anomaly interpretation and reduce false-positive detections.

### 5.7. Future Work

Several directions may further enhance the proposed framework. First, future studies should evaluate the methodology using longer longitudinal datasets spanning multiple months or years to investigate seasonal effects, long-term behavioral drift, and changes in routine over time.

Second, adaptive anomaly thresholds should be explored to accommodate evolving behavioral patterns. Although the 95th percentile threshold provided a transparent rule in the present study, adaptive thresholding strategies should be investigated to better balance sensitivity to gradual routine changes with the risk of excessive false alerts. Future research should also investigate semi-supervised, self-supervised, and unsupervised learning approaches using larger collections of unlabeled smart-home data from older adults. Since clinically validated anomaly labels are rarely available in long-term residential monitoring studies, learning strategies that reduce dependence on manual annotation may support future scalability and practical deployment, but this requires validation in larger and longer-term datasets.

Third, additional machine learning architectures should be investigated and compared with the GRU model. Transformer-based networks, attention mechanisms, temporal convolutional networks, and hybrid deep-learning models may provide improved forecasting accuracy and enhanced anomaly detection capabilities [35,36].

Fourth, future research should examine multi-modal sensing environments that combine motion and contact sensors with wearable devices [3,7], environmental sensors, physiological monitoring systems, and energy-consumption data. Such systems may eventually support predictive healthcare monitoring, personalized risk assessment, simulation of behavioral changes, and early intervention planning, provided that future studies incorporate clinical validation and contextual interpretation. Future research should also investigate the computational efficiency and deployment feasibility of the proposed framework on resource-constrained edge devices commonly used in smart-home environments. Evaluating training time, inference latency, memory consumption, and energy requirements will provide important insights into the practical applicability of the framework for continuous real-time behavioral monitoring.

Another promising direction is the integration of the proposed framework within Digital Twin architectures for older adults [4,37]. A behavioral Digital Twin could continuously update a virtual representation of an individual’s daily routine using real-time sensor observations. Such systems may enable predictive healthcare monitoring, personalized risk assessment, simulation of behavioral changes, and early intervention planning. The combination of smart-home sensing, machine learning, and Digital Twin technologies represents an emerging research area with significant potential for ambient assisted living applications.

Finally, user-centered evaluations involving caregivers, clinicians, and healthcare professionals should be conducted to assess the practical utility of radar-based visual analytics. Understanding how visual behavioral profiles influence decision-making, trust, and intervention planning represents an important step toward real-world deployment of explainable smart-home monitoring systems.

## 6. Conclusions

This study developed and evaluated an interpretable framework for personalized routine-deviation detection in smart-home environments using unlabeled sensor data collected from four independently living older adults. The proposed framework integrates participant-specific spatial-zone selection, GRU-based routine prediction, adaptive participant-specific thresholding, baseline forecasting comparison, and radar-based visual interpretation within a unified workflow. The cross-validation results demonstrate that optimal behavioral representations vary significantly across participants, confirming that individual living habits dictate which architectural zone yields the strongest predictive signal and underscoring the necessity of personalized baselines over global thresholds. Furthermore, the empirical finding that single-zone configurations consistently outperform multi-zone combinations suggests that expanding spatial inputs introduces cumulative behavioral noise that degrades forecasting accuracy.

The proposed GRU model consistently outperformed the Previous-Day baseline for all participants and achieved lower prediction errors than the Seasonal Naïve baseline for three of the four participants, demonstrating the effectiveness of the proposed forecasting framework. The radar visualizations provided qualitative support for interpreting the detected candidate routine-deviation days by illustrating changes in the temporal distribution of activities after detection had occurred. Because the framework relies exclusively on non-intrusive motion and contact sensors, it provides an interpretable and privacy-preserving approach for long-term behavioral monitoring without requiring manually annotated training data.

Although the present study represents a proof-of-concept evaluation involving four participants, the findings demonstrate the feasibility of integrating participant-specific forecasting, adaptive thresholding, and visual interpretation within a single routine-deviation detection framework. Future work will evaluate the proposed methodology using larger participant cohorts, clinically annotated datasets, and additional forecasting architectures to further assess its applicability in independent living support and smart-home healthcare monitoring.

## Figures and Tables

**Table 1:** Overview of deployed sensors and monitored behavioral activities.

Zone	Sensor-Event Used	Zone Equation in Words	Behavioral Meaning
Kitchen/Living	motion_motion1_event contact_doorcontact2_open_event; contact_doorcontact2_close_event	Motion 1 + door-contact 2 open + door-contact 2 close	Movement and door interaction in kitchen/living area
Outdoor	contact_doorcontact1_open_event; contact_doorcontact1_close_event	Door-contact 1 open + door-contact 1 close	Entrance door use and possible outdoor mobility
Bedroom	motion_motion2_event	Motion 2 only	Bedroom movement or occupancy
Washroom	motion_motion3_event contact_doorcontact3_open_event; contact_doorcontact3_close_event	Motion 3 + door-contact 3 open + door-contact 3 close	Washroom movement and door interaction

**Table 2:** Motion sensor dataset statistics.

Users	Total Events	Unique Time Stamps	Number of Sensors	Occupancy True%	Occupancy False%	Duration Days	Events per Day
User1	10,864	10,776	3	42.35	57.65	28	388
User2	40,242	39,550	3	55.94	44.06	28	1437.21
User3	18,914	18,470	3	51.13	48.87	28	675.5
User4	41,366	40,358	5	57.1	42.9	28	1477.36

**Table 3:** Contact sensor dataset statistics.

Users	Total Events	Unique Events	Number of Sensors	Contact True %	Contact False %	Duration Days	Events per Day
User 1	3567	2832	3	75.11	24.89	28	101.14
User 2	8177	8177	3	62.61	37.39	28	292.04
User 3	3523	3523	3	71.96	28.04	28	125.82
User 4	4316	4315	3	65.24	34.76	28	154.11

**Table 4:** Forecasting performance comparison between the proposed GRU framework and simple forecasting baselines under five-fold time-series cross-validation.

User	Target Zone	Model	RMSE	MAE
User 1	Bedroom	Proposed GRU	0.180	0.136
		Previous-Day Baseline	0.229	0.185
		Seasonal Naïve Baseline	0.265	0.211
User 2	Outdoor	Proposed GRU	0.136	0.126
		Previous-Day Baseline	0.238	0.196
		Seasonal Naïve Baseline	0.250	0.224
User 3	Bedroom	Proposed GRU	0.170	0.138
		Previous-Day Baseline	0.351	0.302
		Seasonal Naïve Baseline	0.161	0.145
User 4	Outdoor	Proposed GRU	0.147	0.134
		Previous-Day Baseline	0.222	0.199
		Seasonal Naïve Baseline	0.413	0.359

## Data Availability

The data are not publicly available due to privacy and ethical restrictions.
